# Intermittent neurologic decompensation: An underrecognized presentation of tyrosine hydroxylase deficiency

**DOI:** 10.1002/jmd2.12306

**Published:** 2022-06-06

**Authors:** Marjolaine Champagne, Gabriella A. Horvath, Sébastien Perreault, Julie Gauthier, Keith Hyland, Jean‐François Soucy, Grant A. Mitchell

**Affiliations:** ^1^ Division of Medical Genetics, Department of Pediatrics, Molecular Diagnostic Laboratory, Centre Hospitalier Universitaire Sainte‐Justine Université de Montréal Montréal Québec Canada; ^2^ Division of Biochemical Diseases, Department of Pediatrics BC Children's Hospital Vancouver British Columbia Canada; ^3^ Division of Child Neurology, Department of Pediatrics Centre Hospitalier Universitaire Sainte‐Justine Montréal Québec Canada; ^4^ Integrated Centre for Pediatric Clinical Genomics, Centre Hospitalier Universitaire Sainte‐Justine Montréal Québec Canada; ^5^ MNG Laboratories (Medical Neurogenetics, LLC.), MNG, a Wholly Owned Subsidiary of Laboratory Corporation of America Holdings San Diego California USA

**Keywords:** neurological decompensation, TH, tyrosine hydroxylase deficiency

## Abstract

Tyrosine hydroxylase deficiency (THD) is a treatable inborn error of dopamine biosynthesis caused by mutations in *TH*. Two presentations are described. Type A, milder, presents after 12 months of age with progressive hypokinesis and rigidity. Type B presents before 12 months as a progressive complex encephalopathy. We report a girl with mild THD who had recurrent episodes of neurological decompensations. Before the first episode, she had normal development except for mild head tremor. Episodes occurred at 12, 19, and 25 months of age. After viral infections or vaccination, she developed lethargy, worsened tremor, language, and motor regression including severe axial hypotonia, recuperating over several weeks of intensive rehabilitation but with residual tremor and mild lower limb spasticity. Basal ganglia imaging was normal. Exome sequencing revealed two missense variants of uncertain significance in *TH*: c.1147G>T and c.1084G>A. Both have low gnomAD allele frequencies and in silico, are predicted to be deleterious. Cerebrospinal fluid analysis showed low homovanillic acid (HVA, 160 nmol/L, reference 233–938) and low HVA/5‐hydroxyindolacetic acid molar ratio (1.07, reference .5–3.5). She responded rapidly to L‐Dopa/carbidopa without further episodes. Literature review revealed four other THD patients who had a total of seven episodes of marked hypotonia and motor regression following infections, occurring between ages 12 months and 6 years. All improved with L‐Dopa/carbidopa treatment. Intermittent THD is treatable, important for genetic counseling, and should be considered after even a single episode of marked hypotonia with recuperation over weeks, especially in patients with preexisting tremor, dystonia, or rigidity.

## 1 INTRODUCTION

Tyrosine hydroxylase deficiency (THD, OMIM 191290) is a rare treatable inborn error of dopamine biosynthesis. It is an autosomal recessive neurometabolic disorder due to mutations in the *TH* gene on chromosome 11p15.5.^1^, ^2^, ^3^ More than 60 known pathogenic variants are described in *TH* and its promoter region (HGMD, LOVD). Pathogenic variants decrease TH enzymatic function.^4^ In mice, complete absence of TH activity is lethal.^5^

TH catalyzes the conversion of tyrosine (l‐tyrosine) to levodopa (L‐Dopa), the first step of the biosynthesis of catecholamine neurotransmitters (dopamine, norepinephrine and epinephrine). TH is mainly expressed in dopaminergic and noradrenergic neurons^6^ and in adrenal medulla.^2^, ^7^ Chronic deficiency of dopamine and other catecholamines is felt to underlie the clinical manifestations of this disease. THD can be diagnosed by demonstrating low levels in cerebrospinal fluid of two products of catecholamine degradation, homovanillic acid (HVA) and 3‐methoxy‐4‐hydroxyphenylethylene glycol (3‐MHPG),^8^ and by molecular analysis of *TH*.

Clinical descriptions of THD have identified two clinical categories, differing in severity and response to L‐Dopa.^9^ Type A THD, the milder form, usually presents after 12 months of age with progressive hypokinesis, rigidity and dystonia and responds well to L‐Dopa.^10^ Type B THD usually presents between birth to 12 months of age as a progressive complex encephalopathy and is relatively resistant to L‐Dopa.^10^ Type B THD patients may develop paroxysmal episodes of lethargy, irritability, and oculogyric crises. Usually, Type B patients have lower levels of HVA in cerebrospinal fluid (CSF, 5–50 nmol/L) than Type A patients (23–158 nmol/L).^8^ Typically, both forms evolve in a chronic progressive fashion.

## 2 CASE REPORT

A 36‐month‐old girl had three episodes of neurological decompensation following infections or vaccinations. She is the only child of a healthy nonconsanguineous couple of German, Italian, and Irish descent. Fetal ultrasonography showed a single umbilical artery, a type II atrial septal defect, aberrant right subclavian artery and nonobstructive cor triatriatum. Amniocentesis was declined. Noninvasive prenatal testing showed low risk for trisomies 21, 18, and 13. Labor was induced at 41 weeks of gestation. She was born by vaginal delivery. Birth weight was 3389 g (28th centile); length, 55 cm (93th centile); and head circumference, 34.5 cm (24th centile). She received phototherapy for 24 hours for neonatal jaundice. Prior to the first episode, she had normal development except for mild head tremor. By 12 months, she walked with support, was stable in the sitting position, crawled up stairs, and had a pincer grasp and a vocabulary of several words.

The first episode of deterioration occurred at 12 months of age. Two weeks earlier she had been vaccinated, followed by intermittent fever. She developed marked fatigue, loss of walking/sitting, tremor (appendicular and worsened head tremor), and severe axial hypotonia. She was hospitalized for 3 days and recuperated completely over several days.

Episodes 2 (19 months) and 3 (25 months) each followed viral infections by a few days. Both presented as lethargy, axial hypotonia, appendicular rigidity, and worsening tremor with motor and language regression. After each, she required several weeks of intensive rehabilitation.

Between episodes, examination revealed mild axial tremor, mild lower limb spasticity, and axial hypotonia. She regained her developmental milestones but had a mild motor and language delay after the second episode. At the age of 3 years, neurodevelopmental evaluation revealed a 6 month delay.

**FIGURE 1:**
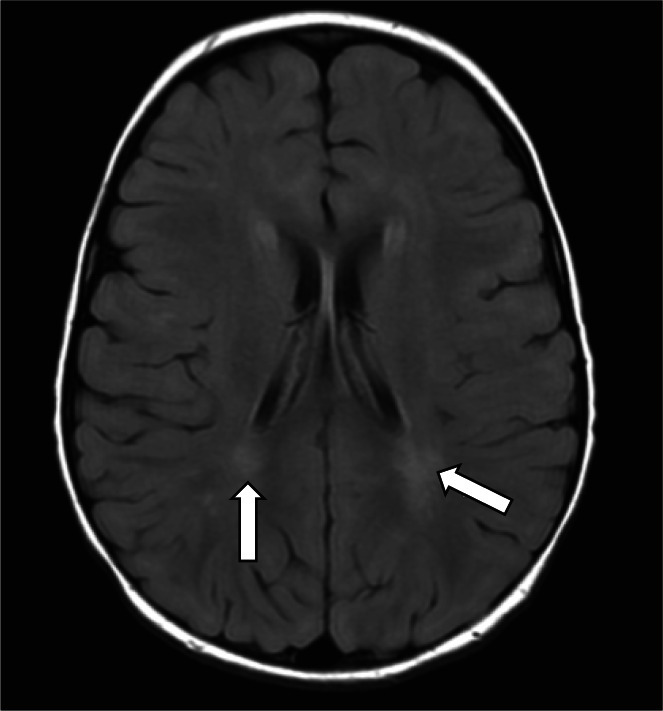
Cerebral MRI of patient 5 at 19 months of age. Axial T2‐FLAIR image showing nonspecific T2‐hyperintense patches in the occipital regions

During each episode, multiple investigations were performed. The following were normal: blood lactate, ammonia, alanine aminotransferase, bilirubin, plasma amino acids, plasma acylcarnitines and urinary purine and pyrimidine derivatives. Initially, she had mild persistent methylmalonic aciduria and low levels of vitamin B12, both of which normalized after oral vitamin B12 replacement. In fibroblasts, assay of galactocerebrosidase, arylsulfatase A, β‐galactosidase, respiratory chain complex activities, and blue native gel analysis were normal. Array CGH, sequencing of *SUCLA2*, *SUCLG1*, and molecular testing for Aicardi‐Goutières syndrome were normal. Cerebral magnetic resonance imaging (MRI) at 19 months (Figure 1) showed nonspecific T2‐hyperintense patches in the occipital and frontal regions that were stable on repeat MRI at 2 years 6 months. Neuroophthalmological examination, audiogram, abdominal ultrasound, electroencephalogram, electromyogram, and nerve conduction studies were normal. The working clinical diagnosis was Leigh‐like syndrome.

Exome sequencing was performed and analyzed at CHU Sainte‐Justine using a standard Illumina® workflow, an in‐house bioinformatics pipeline based on GATK and Annovar. This revealed biallelic missense variants of unknown significance in *TH*: NM_000360.3:c.1147G>T (p.Gly383Trp), inherited from the father and c.1084G>A (p.Glu362Lys), inherited from the mother. Both variants have allele frequencies of <1/50 000 (2 alleles and 3 alleles, respectively, in gnomAD). Both are predicted to be deleterious by SIFT, PolyPhen and MutationTaster. Both are located in the catalytic domain of *TH*.^11^ Neither was previously reported to our knowledge.

Lumbar puncture at 2 years and 11 months revealed that the dopamine metabolite homovanillic acid (HVA) was 160 nmol/L, 32% below the lower reference limit for age (reference, 233–938). 5‐Hydroxyindolacetic acid (5‐HIAA) was normal (150 nmol/L, reference range, 74–345) and the HVA/5‐HIAA molar ratio was low (1.07, literature reference range,1.5–3.5),^12^ compatible with the diagnosis of mild THD.

Dopa/carbidopa treatment (1.7 mg/kg/d of L‐Dopa), initiated immediately after the lumbar puncture, was followed within days by clinical improvement and the patient has had no further episodes. She walked independently at the age of 3 years 3 months. She is now 5 years old and receives 2.7 mg/kg/day of L‐Dopa, divided into four doses. Dyskinesia was detected when doses were increased, needing very slow and gradual dose adjustments. Parents and caregivers have noted some “on and off” phenomena at the end of dosing intervals, which is treated by a MAO inhibitor (Selegiline, 0.5 mg/day in the morning). We chose this approach instead of further increasing the L‐dopa dose, because of concerns of potential dopamine supplementation‐related dyskinesia. At 4 years of age, her general physical examination was normal. Neurologically, her pattern of running was still somewhat immature, but she was stable without falls. She had mild axial hypotonia. Deep tendon reflexes were normal and symmetrical. She could heel‐ and toe‐walk. She had no dystonia or abnormal posturing. She had mild tremor on finger‐to‐nose testing and minor brief dyskinetic movements that did not interfere with daily functioning.

## 3 LITERATURE REVIEW

### 3.1 Methods

We reviewed the medical literature for THD patients with episodic neurologic decompensations. We last searched PubMed on 30/08/2020 for English‐language articles with the following interrogation: “Dopa‐responsive dystonia” OR “Infantile hypokinetic rigid Parkinsonism” OR “Tyrosine hydroxylase deficiency” OR “Segawa's disease.” From these articles and their references, we attempted to identify other articles. We summarized the clinical, genetic, and biochemical features of patients with episodic decompensations or crises (Table 1).

### 3.2 Results

**FIGURE 2:**
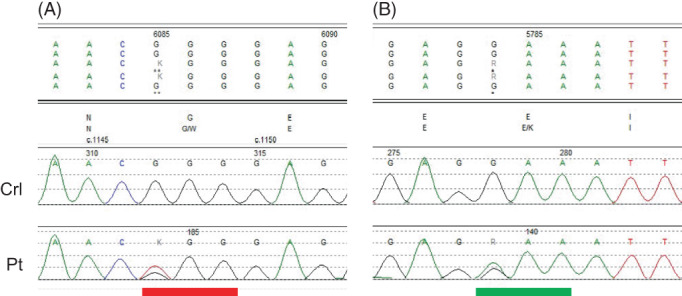
Sanger sequencing analysis of the *TH* variants in patient 5. The affected codons are indicated. (A) c.1147G>T (p.Gly383Trp). (B) c.1084G>A (p.Glu362Lys). Crl, control; Pt, patient

Including our patient, we identified five individuals with mild THD who presented at least one acute neurologic decompensation (Table 1 and Figure 2). A total of 10 distinct neurologic crises were described sufficiently for inclusion. All crises occurred after infections or vaccinations, between 12 months and 6 years of age. Infections included viral infection with fever, pneumonia, and cervical lymphadenopathy. Most patients had neither neurologic symptoms (3/5) nor developmental delay (4/5) before the first episode. Two patients (3 and 5) had tremor before their first decompensation.

**TABLE 1:** Clinical features, genetic findings, and CSF neurotransmitter metabolite levels of THD patients with intermittent neurologic crises^1^, ^12^, ^14^, ^15^

Patients	1	2	3	4	5	Range or fraction (%)
	Diepold, 2005^12^	Yeung, 2011^14^	Haugarvoll, 2011^14^	Katus, 2017^1^	This report	
Family origin	Europe	China	NR	Myanmar	Europe	
Sex	F	M	M	M	F	4 M, 2 F
Age at clinical onset	14 months	3 years	14 months	4 years	12 months	12 months–4 years
Diagnosis of THD (age)	2 years 4 months	16 years	25 years	31 years	2 years	2–31 years
Number of crises	2	1	2	2	3	1–3 crises/patient
Age at crises	14 months, 19 months	3 years	6 years	4 years	12, 19, and 25 months	12 months–6 years
Identified precipitant	+ (I)	+ (I)	+ (I)	+ (I)	+(I, V)	5/5 (100)
Neurologic signs before first crisis	−	−	+ (tremor)	−	+ (tremor)	2/5 (40)
*Neurologic features during acute crises*						
Dystonia	+	−	+	−	−	2/5 (40)
Hypo/bradykinesia	+	+	−	+	+	4/5 (80)
Tremor	+	+	+	−	+	4/5 (80)
Hypotonia	+	−	+	+	+	4/5 (80)
Rigidity/hypertonia	+	−	+	−	+	3/5 (60)
Lethargy	+	+	+	−	+	4/5 (80)
Loss of milestones	+	+	+	+	+	5/5 (100)
*Developmental profile*						
Normal cognitive development before first episode	+	+	− (Delay)	+	+	4/5 (80)
Motor delay	+	−	+	−	+	3/5 (60)
Intellectual disability	ND	−	−	−	ND	0/3 (0)
*CSF/Imaging findings*						
HVA low (patient, reference range [nmol/L])	+ (126, 211–871)	+ (110, 115–488)	ND	ND	+ (160, 233–928)	3/3 (100)
5‐HIAA normal (patient, reference range [nmol/L])	+ (339, 105–299)	+ (100, 66–141)	ND	ND	+ (150, 74–345)	3/3 (100)
HVA/5‐HIAA ratio low (patient, reference range)	+ (0.37, 1.5–3.5)	+ (1.1, 1.5–3.5)	ND	ND	+ (1.07, 1.5–3.5)	3/3 (100)
Normal cerebral MRI	+	+	+	ND	−	3/4 (75)
*Molecular variants*						
cDNA (aligned to NM_000360.3)	c.643C>T/c.1400A>G	c.364C>T/c.1061C>T	c.1229G>C/c.1400A>G	c.1388C>T	c.1147G>T/c.1084G>A	
Protein	p.His215Tyr/p.Asp467Gly	p.Arg122*/p.Ala354Val	p.Arg410Pro/p.Asp467Gly	p.Thr463Met	p.Gly383Trp/p.Glu362Lys	
*Clinical course after treatment with L‐dopa/carbidopa*						
Minimal residual neurologic features	+	−	+	+	+	4/5 (80)
Further crises	−	−	−	−	−	0/5 (0)

*Note*: Loss of milestones, includes loss of walking, standing, sitting, and/or speaking.

Abbreviations: 5‐HIAA, 5‐hydroxyindolacetic acid; F, female; HVA, homovanillic acid; I, infection; M, male; ND, Not done; NR, not reported; V, vaccination.

Each crisis was accompanied by marked motor regression, often with loss of walking, standing and/or sitting. Loss or reduction of speech was frequent. The principal neurologic symptoms were tremor (4/5), hypo/bradykinesia (4/5), hypotonia (4/5), and lethargy (4/5). Complete or partial recuperation occurred in all cases after episodes.

Two patients were diagnosed during infancy (two and two and a half years). Three were diagnosed between 16 and 31 years of age. Biochemical findings in the CSF are summarized on Table 1.

**FIGURE 3:**
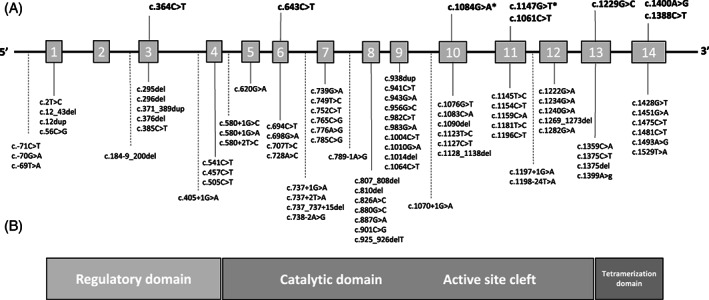
*TH* gene structure and reported pathogenic variants in THD patients with intermittent and chronic clinical courses. The *TH* gene is drawn according to reference sequence NM_000360.3. Exons 1–14 are shown schematically.^16^, ^17^ Variants described in THD are shown: above the gene diagram, variants in patients with THD and intermittent signs; below, variants described in chronic TH deficiency (forms A and B). The functional domains of the TH protein^7^ are shown at the bottom. *, likely pathogenic variants in patient 5

All five patients had different *TH* gene variants, except that c.1400A>G was present in patients 1 and 3. All variants except one were missense. Patient 2 had a frameshift variant on one allele. The locations and type of *TH* variants in intermittent THD do not stand out clearly from those reported in Types A and B patients with chronic progressive courses (Figure 3).

No patient developed crises after the start of L‐DOPA treatment. Although four patients had detectable neurological motor signs after treatment, outcome in all patients was encouraging and cognitive function was generally normal. Patients 1, 4, and 5 had only minor neurologic symptoms after treatment. Patient 1 had hypotonia of the trunk and mild developmental delay, patient 4 had dystonia of the neck and trunk while walking and patient 5 had mild tremor and mild axial hypotonia. Patient 2, who was treated at 25 years of age, had more significant neurological signs including walking with a crutch and persistent dystonia that was improved with treatment.

## 4 DISCUSSION

These five patients define an important but previously under‐recognized category of THD, featuring episodes of marked hypotonia, in which complete recuperation of muscle tone is possible over weeks to months. Intermittent THD should be considered after even a single unexplained episode of marked hypotonia and loss of milestones. Patients may be asymptomatic between episodes or have chronic mild neurologic findings such as dystonia, hypotonia, motor delay, spasticity and especially, tremor. Chronic tremor was present before the crises in two patients.

The patients described here differ markedly from types A and B TDH, which show chronic progressive courses. Available clinical evidence suggests that intermittent THD should be considered as a third category of THD, distinct from typical descriptions of both Type A (progressive hypokinesis, rigidity, and dystonia presenting after 12 months of age, often showing L‐Dopa responsiveness), and Type B (congenital or infantile‐onset encephalopathy, sometimes with paroxysmal episodes of lethargy, irritability, and oculogyric crises, with or without L‐Dopa responsiveness). Future clinical series should reveal whether these three categories are distinct or merge into a continuum.

Neurologic decompensation with normal systemic metabolic testing results can occur in many neurologic diseases, including several inborn errors of metabolism.^13^ As illustrated in this article, THD must also be considered in such patients. Whole exome sequencing lead to the diagnosis in patient 5. CSF analysis is recommended even if molecular testing is negative, because some pathogenic variants may not be recognized by molecular testing. Furthermore, CSF analysis can clarify molecular findings, as in this report, in which low levels of HVA and of the HVA/5‐HIAA ratio, strongly support the pathogenicity of the two previously unreported *TH* variants, c.1147G>T and c.1084G>A.

For completeness and for future clinical series, we note that patient 5 has a hemodynamically insignificant form of the rare heart malformation, cor triatriatum. This finding has no obvious relationship to THD.

There is practical importance in expanding the clinical classification of THD to include an intermittent form that is mild in comparison to types A and B, because all five such patients identified to date responded to L‐Dopa treatment. For patients with intermittent presentations, the diagnosis of THD might not be considered from current descriptions of this condition in chapters and reviews. Identification of an intermittent form of THD will therefore facilitate early diagnosis for patients with intermittent manifestations. By extension, because of the L‐Dopa responsiveness of the intermittent THD patients known to date, this may help to reduce the risk of neurologic disability in affected individuals.

## CONFLICT OF INTEREST

The authors declare that there is no conflict of interest.

## ETHICS STATEMENT

All procedures followed were in accordance with the ethical standards of the responsible committee on human experimentation (institutional and national) and with the Helsinki Declaration of 1975, as revised in 2000 (5). Informed consent was obtained from all patients for being included in the study.

## Data Availability

The manuscript is a medical case report and literature review. The data in this article are taken from the patient's medical chart. For the literature review, they are taken from the publications referenced. Because of confidentiality, the original data in the patient's chart are not publically available.
